# Two novel formulae are superior to procalcitonin for prediction of sepsis in trauma patients

**DOI:** 10.1186/cc14013

**Published:** 2014-12-03

**Authors:** H-p Liang, H Jin, Y Xiao, Z Liu

**Affiliations:** 1 State Key Laboratory of Trauma, Burns and Combined Injury, Research Institute of Surgery, The Third Military Medical University, Chongqing, People's Republic of China

## Introduction

The purpose of this study was to verify the predictive value of two novel formulae and compare them with that of procalcitonin (PCT) for predicting sepsis in trauma patients.

## Methods

We performed a retrospective study of trauma patients treated at Daping Hospital in Chongqing, China and Affiliated Hospital of Zunyi Medical College between 2010 and 2013. Patients ≥16 years old, admitted to hospital after injury within 24 hours and with length of hospital stay ≥48 hours were included. Predictive ability of two formulae based on LD_50 _values of the Injury Severity Score (ISS) and New Injury Severity Score (NISS) were verified: ISS/LD_50_ISS+SIRS score and NISS/LD_50_NISS+SIRS score, and then were compared with the most common used biomarker PCT. LD_50 _values for different age groups and genders were obtained in our former study. The statistical performance of the two formulae and PCT to predict sepsis was evaluated using receiver operating characteristic (ROC) curve analysis.

## Results

Two hundred and twenty-one trauma patients' data were enrolled in the study, including 44 females and 177 males. The average age of the patients was 44.77 ± 15.01 years. The performance of the ISS/LD_50_ISS+SIRS score and the NISS/LD_50_NISS+SIRS score was equivalent (area under the ROC curve (AUC) = 0.816 vs. 0.819, *P *>0.05) and both performed better than PCT (AUC = 0.592, *P *< 0.05) in predicting posttraumatic sepsis. For the ISS/LD_50_ISS+SIRS score, the cutoff value was 2.38, with a positive predictive value of 78.08%, a negative predictive value of 81.33%, a sensitivity of 89.06%, a specificity of 65.59%, a positive likelihood ratio of 2.59, a negative likelihood ratio of 0.17, a Youden index of 0.5465, an odds ratio of 15.52, and an accuracy of 79.19%. For the NISS/LD_50_NISS+SIRS score, the cutoff value was 2.4677, with a positive predictive value of 79.70%, a negative predictive value of 75.00%, a sensitivity of 82.81%, a specificity of 70.97%, a positive likelihood ratio of 2.85, a negative likelihood ratio of 0.24, a Youden index of 0.5378, an odds ratio of 11.78, and an accuracy of 77.83%.

## Conclusion

The two novel formulae ISS/LD_50_ISS+SIRS score and NISS/LD_50_NISS+SIRS score performed well and were both better than PCT in predicting sepsis post trauma. The value of the two formulae can be easily calculated in real time and can identify the high-risk patients susceptible to sepsis. This method may become an effective way to guide the early assessment and treatment in trauma patients.

